# Acute Appendicitis following Laparoscopic Live Donor Nephrectomy

**Published:** 2010-05-01

**Authors:** A. Kumar, H. Elenin, C. Clayton, C. Basarab-Horwath, B. Man Shrestha

**Affiliations:** Sheffield Kidney Institute, Sheffield, UK

**Keywords:** Appendicitis, Laparoscopic donor nephrectomy, Kidney donor

## Abstract

Acute abdominal pain following laparoscopic live donor nephrectomy (LLDN) might be a diagnostic dilemma, and prompt diagnosis and management is of paramount importance. Herein, we describe a case of acute appendicitis in a 62-year-old kidney donor who presented with acute abdominal pain 16 days following LLDN with features inconsistent with a diagnosis of acute appendicitis. An ultrasound scan suggested strangulated Spigelian hernia unrelated to the operative wound. Exploration of the wound and mini-laparotomy showed no evidence of wound dehiscence or a hernia, but revealed an inflamed appendix wrapped up with omentum. Appendectomy led to complete recovery of the patient. It is imperative to maintain a high index of suspicion for acute appendicitis in this situation to avoid septic complications that might adversely affect the residual renal function and cause negative impact on kidney donation. To the best of our knowledge, this is the first reported case of acute appendicitis following LLDN.

## INTRODUCTION

Increasing number of laparoscopic live donor nephrectomies (LLDN) are being performed worldwide. LLDN decreases the disincentives for live kidney donation significantly due to reduced post-operative pain and shorter recuperation time [1]. Post-operative complications prolong hospital stay, recovery and time to return to work [2]. This has significant implications on the psychological aspects of both donor and recipient and can make negative impact on kidney donation. Herein, we report the presentation and management of a case of acute appendicitis following hand-assisted LLDN (HLLDN). To the best of our knowledge, this is the first report of acute appendicitis occurring after live kidney donation in English literature.

## CASE PRESENTATION

A 62-year-old Caucasian male underwent transperitoneal left HLLDN, where access to the peritoneal cavity was gained through a 9-cm long infraumbilical transverse incision. The skin and the anterior rectus sheath were divided transversely and the peritoneum was opened vertically in the midline. Two 12-mm ports were inserted over the left upper abdomen. A pneumoperitoneum was maintained by insufflating carbon-dioxide (CO_2_) at a pressure between 8 and 12 mm Hg. A routine laparoscopy revealed no abnormalities within the peritoneal cavity. Mobilization of the left colon, ureter, renal vessels and kidney was performed and the blood vessels were divided using ETS-Flex 45 Endo GIA Linear Cutter (Ethicon Inc, Somerville, NJ, USA). The wound was closed in layers with absorbable sutures. The post-operative course was uneventful and he was discharged to home after two days.

Sixteen days following the nephrectomy, he presented with severe pain over the right side of the infraumbilical wound. This was not associated with any fever, loss of appetite, vomiting or change in bowel or bladder habits. On physical examination, he had no fever and was hemodynamically stable. There was a localized deep tenderness near the right margin of the wound. There was no palpable mass, cough impulse nor any sign of peritonism.

**Figure 1:**
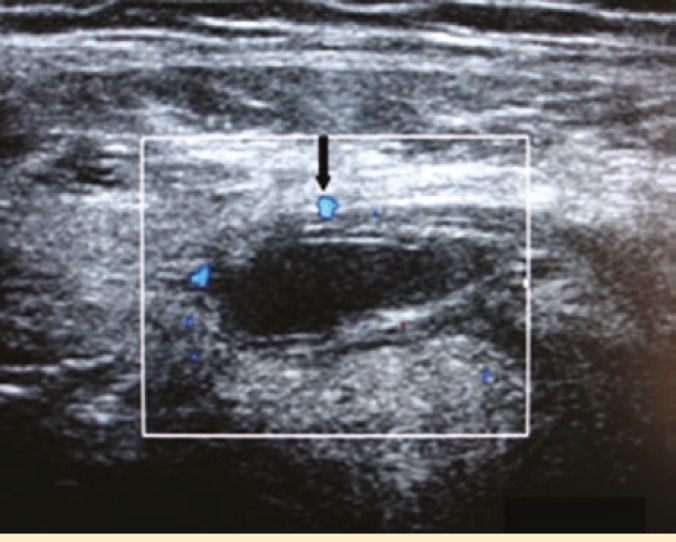
Ultrasound scan showing hypoechoic lesion lateral to the rectus abdominis

The complete blood count and renal function tests were within normal limits (hemoglobin: 13.2 g/dL, white blood cell count: 8.8×10^6^/L; platelet count: 562×10^6^/L and serum creatinine: 138 µM/L). An ultrasound scan revealed a 16×5 mm hypoechoic lesion protruding into the space between the abdominal muscles at the lateral edge of the *rectus abdominis* in the right iliac fossa. Doppler sonography indicated blood flow within the mass; however, there were no visible peristalsis (Fig. 1). The lesion was reported as a Spigelian hernia or a wound dehiscence containing omentum or extraperitoneal fat.

Under general anesthesia, the lesion was explored through a transverse incision over the most tender site marked preoperatively. The muscles were split lateral to the *rectus abdominis* muscle. No hernia could be detected. On palpation over the intact peritoneum, an ill-defined lump was felt, hence, the peritoneum was opened. There was some turbid fluid around an inflamed appendix, which was wrapped up by the omentum. Appendectomy was carried out in the conventional manner. Histopathologic study confirmed the diagnosis of acute appendicitis. The post-operative period was uneventful. Eight months later, the patient presented with a left inguinal hernia which was treated with a mesh repair.

## DISCUSSION

Complications following LLDN such as injury to the spleen and intestine, bleeding, rhabdomyolysis, chylous ascites and intestinal obstruction from internal hernia have been described previously [3, 4]. Acute appendicitis, *per se* has no relationship with LLDN. However, the possibility of strangulation of the appendix through a defect on the wound could be considered, which was not among the operative findings in our case. The other predisposing factors such as fecolith, atherosclerosis, and obstructing lesions at the base of the appendix were absent. Constipation, which is related to mobilization of the colon and side-effects of analgesics, is a common observation following LLDN. This could predispose to acute appendicitis [5]. There was no intra-operative hypotension in the donor to cause arterial thrombosis in the appendix. As far as the effects of laparoscopy on the immune system is concerned, studies have shown better preservation of systemic immunity after laparoscopic compared to open surgery [6, 7].

In our case, the diagnostic dilemma caused by the atypical clinical presentation following HLLDN remained unresolved until the appendicitis was confirmed at operation. The ultrasound scan had shown a mass in the intramuscular plain containing omentum or extraperitoneal fat, which prompted the diagnosis of a strangulated Spigelian hernia or wound dehiscence. A computed tomography was not carried out deliberately to avoid contrast-induced nephropathy in the solitary kidney. 

Septic complications related to appendicitis can seriously affect the residual renal function on the remaining kidney after nephrectomy. This case emphasizes the fact that acute appendicitis should be considered in the differential diagnosis of acute abdominal pain in a perfectly healthy live kidney donor after undergoing LLDN even if the presentation may not be typical. A prompt diagnosis and management is of paramount importance as mismanagement can lead to serious consequences and cause negative impact on kidney donation.

## References

[1] Hamidi V, Andersen MH, Oyen O (2009). Cost effectiveness of open versus laparoscopic living-donor nephrectomy. Transplantation.

[2] Nanidis TG, Antcliffe D, Kokkinos C (2008). Laparoscopic versus open live donor nephrectomy in renal transplantation: a meta-analysis. Ann Surg.

[3] Knoepp L, Smith M, Huey J (1999). Complication after laparoscopic donor nephrectomy: a case report and review. Transplantation.

[4] Breda A, Veale J, Liao J, Schulam PG (2007). Complications of laparoscopic living donor nephrectomy and their management: the UCLA experience. Urology.

[5] Beck A (1954). Constipation: a true symptom in acute appendicitis. Med World.

[6] Duchene DA, Gallagher BL, Ratliff TL, Winfield HN (2008). Systemic and cell-specific immune response to laparoscopic and open nephrectomy in porcine model. J Endourol.

[7] Gupta A, Watson DI (2001). Effect of laparoscopy on immune function. Br J Surg.

